# Conflicts over calcium and the treatment of COVID-19

**DOI:** 10.1093/emph/eoaa046

**Published:** 2020-11-23

**Authors:** Bernard Crespi, Joe Alcock

**Affiliations:** 1 Department of Biological Sciences, Simon Fraser University, Burnaby, BC, Canada; 2 Department of Emergency Medicine, University of New Mexico, Albuquerque, NM, USA

**Keywords:** SARS-CoV-2, COVID-19, conflict, hypocalcemia, calcium

## Abstract

Several recent studies have provided evidence that use of calcium channel blockers (CCBs), especially amlodipine and nifedipine, can reduce mortality from coronavirus disease 2019 (COVID-19). Moreover, hypocalcemia (a reduced level of serum ionized calcium) has been shown to be strongly positively associated with COVID-19 severity. Both effectiveness of CCBs as antiviral therapy, and positive associations of hypocalcemia with mortality, have been demonstrated for many other viruses as well. We evaluate these findings in the contexts of virus–host evolutionary conflicts over calcium metabolism, and hypocalcemia as either pathology, viral manipulation or host defence against pathogens. Considerable evidence supports the hypothesis that hypocalcemia represents a host defence. Indeed, hypocalcemia may exert antiviral effects in a similar manner as do CCBs, through interference with calcium metabolism in virus-infected cells. Prospective clinical studies that address the efficacy of CCBs and hypocalcemia should provide novel insights into the pathogenicity and treatment of COVID-19 and other viruses.

## INTRODUCTION

Therapeutic agents to reduce morbidity and mortality from human infectious diseases work in one of several main ways. The agent may attack the pathogen directly, through interference with the functions of the proteins that it codes for and the nucleic acids that it requires to survive and replicate. The RNA nucleotide analog remdesivir provides a good example of this approach, as it reduces the ability of the severe acute respiratory syndrome coronavirus 2 (SARS-CoV-2) virus to copy its genetic material [1]. Such agents can be highly effective but they are prone to the evolution of resistance, due to the strong selection imposed and the usual high numbers of genetically variable pathogens present in any given infection.

Alternatively, a therapeutic agent may modulate some aspect of the human immune system. For example, the corticosteroid dexamethasone, which dampens inflammatory activity, has been demonstrated in one recent study to reduce mortality in severe cases of coronavirus disease 2019 (COVID-19) [2], which are characterized by high inflammation and associated microvascular damage [3, 4]. This ameliorative effect was found among individuals of mean age 59, but not in more-elderly patients. The use of agents that modify the immune system is predicated on the assumption that the body’s immune reaction is, for some reason, dysregulated in a given disease or patient. Such dysregulation has been well established for COVID-19 [3].

A third domain of agents and tactics used in fighting infectious disease is those deployed by the body itself: the changes in the body that represent its own adaptive responses to infection. Examples of such strategies include sequestration of iron [5], fever [6], other elements of acute-phase responses such as inflammation [7] and adaptive immunity. Such tactics can impose substantial costs on the host as well as on pathogens, but with expected net benefits across a population of infected hosts overall, given that the relevant immune-related pathways have evolved. In severe infection, the body escalates its defences, resulting in higher and higher costs to both the pathogens and itself [8], and more-substantial departures from physiological equilibrium. A clinician may then feel compelled to consider the reaction as pathological, inhibit its effects, and seek to restore a patient to pre-infection levels for the relevant factor. Should they, as a matter of course?

In this article, we describe evidence and theory regarding the use of calcium channel blockers (CCBs) as therapeutic agents against COVID-19. We do so in the broader contexts of host–virus evolutionary conflicts, calcium homeostasis, hypocalcemia (low levels of serum calcium ions) as a host defence, a viral tactic or a pathology of infection, and the main forms of therapy described above.

We first provide an overview of the physiological functions of CCBs and their typical clinical uses in the treatment of hypertension. Second, we discuss recent studies suggesting efficacy of some CCBs in treating COVID-19. Third, we briefly describe how and why viruses depend on ionic calcium. Fourth, we explain the evidence regarding antiviral activity of CCBs, in general and against coronaviruses in particular. This exposition is situated in the more-general framework of how calcium metabolism mediates disease and bodily defences. Fifth, we address the hypothesis that hypocalcemia, a physiological state that typifies COVID-19 and other severe infections, represents a beneficial host defence rather than a pathology or an adaptation of pathogens. Moreover, this host defence may mimic CCBs in its impacts on the metabolism of calcium and its deleterious effects on viruses. Finally, we describe the implications of the results for the treatment of COVID-19.

## CALCIUM CHANNEL BLOCKERS

CCBs are used predominantly to reduce blood pressure in people with hypertension. They function by blocking the calcium channels that regulate contractility of the smooth muscle lining peripheral arteries, thereby causing vasodilation and lowering of blood pressure.

CCBs are categorized into two groups, dihydropyridines and non-dihydropyridines, that differ in chemical structure and their range of effects [9]. Many dihydropyridines, including amlodipine, nifedipine, and other ‘-pines’, are approved for clinical use, as are two non-dihydropryidines, diltiazem and verapamil. All CCBs reduce levels of intracellular calcium, and the latter two agents also reduce cardiac contractility. Dihydropryidine CCBs, especially amlodipine (which has an especially favorable effect profile that includes low retention of fluids), are among the most-commonly prescribed drugs for hypertension worldwide [10].

## CCBS AS TREATMENTS FOR COVID-19

A variety of anti-hypertension medications have been studied for their effects on COVID-19 outcomes. Angiotensin-converting enzyme (ACE) inhibitors (ACEIs) and angiotensin-receptor blockers (ARBs) have been of special interest, because SARS-CoV2 uses ACE2 for viral entry into cells, and these medications upregulate the expression of ACE2 in animal models [11]. However, an observational trial from Italy, published in the *NEJM*, reported no effects of ACEIs or ARBs on COVID-19 outcomes [12], nor any effects from CCBs (as a class) or other blood pressure medications. A study using the Danish national health registry similarly found no effect of ACEIs or ARBs on COVID-19 mortality [13], a conclusion that was also reached in a recent meta-analysis [14].

Three *in vivo* studies, each of them retrospective, have specifically evaluated the efficacy of CCBs against COVID-19.

Zhang *et al*. [15] studied 90 hypertensive patients with COVID-19, of whom 44 had been taking amlodipine, 16 nifedipine, 4 other CCBs, 17 other anti-hypertensive agents and 9 no antihypertensive drug. Case fatality rates were significantly lower in the amlodipine treated group (6.7%, 3 of 44), than in the pooled non-amlodipine group (26.1%, 12 of 46) (*P* < 0.05 with adjustments for age and sex).

Solaimanzadeh [16] studied 65 hypertensive COVID-19 patients, 24 of whom were taking CCBs (amlodipine or nifedipine), with 41 not on CCBs, during hospitalization. Amlodipine or nifedipine use was associated with significantly lower case fatality rates (14.6% vs 50%, *P* < 0.01), and lower rates of mechanical ventilation (4.2% vs 39%, *P* < 0.01).

Reynolds *et al*. [17] studied 2573 COVID-19 patients with histories of hypertension, 634 of whom had severe illness as indicated by intensive care unit (ICU) admission, ventilation, or death. In this analysis, previous use of CCBs was associated with a ‘slightly higher’ (4%) risk of severe illness, which was considered not to be of clinical significance. The specific CCBs used by patients were not described.

A second line of evidence relevant to CCB effects on SARS-CoV-2 is *in vitro* studies. Zhang *et al*. [15] showed that treatment with the CCBs amlodipine or benidipine, but not ACE inhibitors or angiotensin II receptor blockers, showed significant anti-viral effects against SARS-CoV-2 in Vero E6 green monkey cells. Similarly, Straus *et al*. [18] showed, in Vero E6 cells, and in epithelial kidney cells, that amlodipine, felodipine and nifedipine limited the growth of SARS-CoV-2. Hoagland *et al*. [19] demonstrated, using stem-cell derived pancreatic organoids, that the CCBs amlodipine and berbamine reduced levels of viral transcription by about three orders of magnitude; amlodipine also caused selective differential expression of type 1 interferon pathway signaling genes, which play central roles in coronavirus–host interactions.

A third source of evidence, here relevant to effects of amlodipine in treatment of COVID-19, comes from an analysis of the genetic risk factors affecting COVID-19 mortality [20]. Allelic variation at seven single nucleotide polymorphisms has been significantly associated with mortality from COVID-19 infection [21, 22]. A phenome-wide association study, which determines what phenotypes these single-nucleotide polymorphisms (SNPs) have been associated with in previous genome-wide association studies, showed that four of the seven SNPs had been linked with use of amlodipine [20]. These findings suggest, with indirect evidence, that use of amlodipine mediates COVID-19 survival.

These convergent sources of data provide biological plausibility for the potential use of CCBs, and perhaps amlodipine in particular, in the treatment or prevention of COVID-19 (see also [23, 24]). Prospective clinical trials are needed, especially given that hypertension has been reported to be a substantial risk factor for COVID-19 mortality [25].

## VIRUS DEPENDENCE ON IONIC CALCIUM

Ca++ is necessary for viral entry into host cells, viral gene expression, processing of viral proteins, and viral maturation and release [26–31]. To meet their needs for calcium, many pathogenic viruses induce increased influx of these ions across cell membranes (e.g. [29–31]). These influxes of calcium are often mediated by viroporins that act as viral-encoded calcium channels, facilitating calcium input into the cytoplasm from across the cell plasma membrane or across the membrane of the endoplasmic reticulum, which acts as a store for intracellular ionic calcium [32]. In other host–virus systems, viruses use host-encoded cellular calcium channels to increase Ca++ entry into the cytoplasm (e.g. [27]).

Viruses thus cause selective alterations to calcium signaling in host cells as central aspects of their strategies for efficient replication [33, 34], with the alterations normally involving increased intracellular levels [26, 27]. These findings suggest that conflicts between hosts and viruses may frequently involve calcium. Pathogen manipulation of host calcium metabolism also apparently extends to bacterial infection; for example, Bosson *et al*. [35, 36] showed that CCBs improved survival in two animal models of sepsis.

## MECHANISMS OF CCB EFFECTS

How do CCBs work in the context of viral infection, and how might they impact the pathogenicity of COVID-19? Ionic calcium regulates many fundamental intracellular processes through its activity as a second messenger for transduction of signals [26]. Agents that block calcium transport across membranes, such as CCBs, may affect SARS-CoV-2 and the symptoms of COVID-19 by any of several mechanisms.

**Figure 1.**
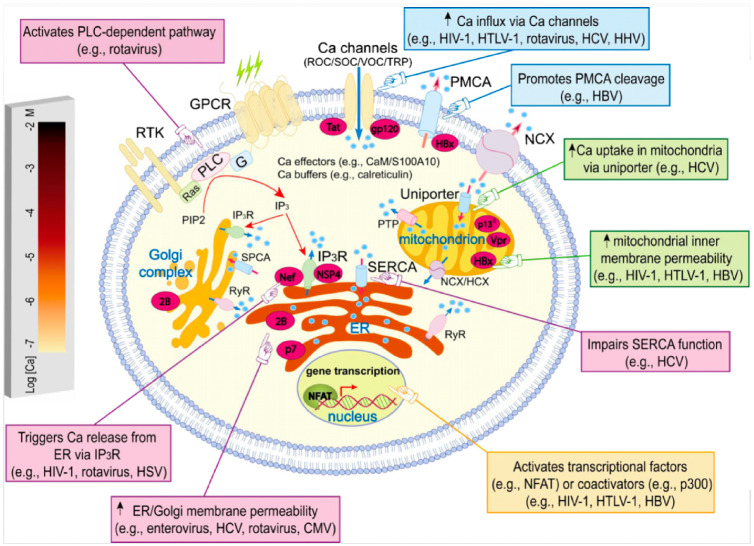
Examples of how viruses disrupt and exploit calcium signaling in host cells. Calcium ions are represented as blue circles. Reprinted from Zhou *et al*. [27] with permission

CCBs reduce levels of intracellular calcium [37], presumably countering this and other calcium-manipulating adaptations of viruses (e. g. [38]). Table 1 illustrates the range of viral manipulation and exploitation of host calcium across diverse viruses, and provides examples of CCB effects that alleviate viral infections through interference with these mechanisms. Examples of specific virus-induced alterations to host calcium metabolism are also illustrated in Fig. 1.

**Table 1.** Examples of Ca++ effects in viral infection, and their inhibition by CCBs or other calcium blocking or reducing agents

Virus	Alterations to Ca++	Comments	Refs.
Porcine coronavirus PDCoV	Infection leads to upregulation of intracellular Ca++ concentrations	Treatment with CCB (diltiazem) inhibited viral replication	31
Murine coronavirus	Infection induced rapid calcium increase in about 5% of cells (apparently those infected by multiple viruses)	CCB verapimil inhibited viral replication	39
SARS CoV and MERS	Ca++ required for viral entry	CCBs inhibit SARS CoV infection *in vitro*	40
Recovirus	Increased cytosolic Ca++ levels mediated by viroporin NS1-2 shown to facilitate viral replication	Virus yield reduced by experimental Ca++ reduction	41
Dengue virus	Infected cells show increased permeability to Ca++, mediated by virus	Ca++ increases favored viral replication; virus yield reduced by Ca++ reduction	42
Hepatitis B virus	HBx protein stimulates Ca++ entry into cells	Reduction of Ca++ impaired viral replication	43
West Nile virus	Infection leads to rapid Ca++ influx into cells, via calcium channels	Treatment with CCBs (verapamil, diltiazem, nifedipine) decreased viral yield	28
Dengue, hepatitus C and Zika	Cellular ion channel TRPV4 mediates Ca++ influx	Blocking of TRPV4 channel reduced viral infectivity	30
Herpes virus	Infection induces rapid and transient increased in intracellular Ca++	Ca++ alteration mediates viral entry into cells	44
Phelbovirus	Infectivity mediated by Ca++ influx into cells	CCBs (benidipine or nifedipine) reduced intracellular Ca++ and improved survival in mouse model and in human retrospective study	45
Influenza A	Infection triggers influx of Ca++	CCBs (verapimil and diltiazem) inhibit viral infection	46–48
Rotavirus	Influx of Ca++ early in infection due to viroporin NSP4	Viroporin-defective mutant lacked Ca++ conductivity	32, 49
Filoviruses (Ebola and Marburg)	Viral entry into cells requires Ca++ permeable ion channel TPC2	Calcium channel blocker verapamil (and other channel blockers) inhibit viral cell entry	50, 51
Coxsackievirus	Influx of Ca++ early in infection due to viroporin 2B	Viroporin with mutations showed low infectivity	32, 52
Cytomegalovirus	Virus induces early influx of Ca++ from extracellular environment, perhaps via viroporin US21	CCBs nifedipine, verapamil and manidipine inhibit virus	53, 54

The coronaviruses SARS-CoV and Middle East respiratory syndrome (MERS)-CoV are known to use calcium ions to orchestrate entry into host cells, via a fusion peptide derived from the spike protein [55, 56]. Experimental depletion of intracellular or extracellular Ca++ (or both) eliminates or reduces viral entry [40, 56]. The close similarity of SARS-CoV and MERS-CoV to SARS-CoV-2 [57] suggests that the same or similar mechanisms apply to the current pandemic virus. SARS-CoV and other coronavirus also produce an envelope (E) protein that can assemble into viroporins, whose activity leads to increased inflammation, damage to host cells and edema [58, 59].

A second mechanism of potential antiviral CCB effects is indicated by the observation that amlodipine and other CCBs exert anti-inflammatory and anti-coagulatory effects in humans [60, 61] and animal or cell models [62–66]. These mechanisms are important because, as noted above, high levels of inflammation and microvascular coagulation are considered as major causes of COVID-19 morbidity and mortality [3, 4].

Third, Solaimanzadeh [16] suggests that the vasodilatory effects of CCBs in the lungs and vascular system may mitigate the effects of high inflammation, hypercoagulation, edema and local vasoconstriction, facilitating oxygen diffusion and host cell survival.

The hypotheses described above for the effects of CCBs in COVID-19 and other viral diseases are not mutually exclusive. Determining which are most relevant has key implications, however, for COVID-19 treatments that involve alterations to the metabolism of calcium in viruses and hosts.

## HYPOCALCEMIA: PATHOLOGY, VIRAL TACTIC OR HOST DEFENCE?

In many viral diseases, concentrations of serum calcium decrease substantially without medical intervention. In particular, many such diseases are characterized by so-called hypocalcemia, defined as serum levels of ionic Ca++ below some threshold. Severe hypocalcemia can cause cramps, numbness, cardiac arrhythmia, seizures, delirium, hypotension and death (e.g. [67]). If it is deleterious to the host, why does hypocalcemia frequently accompany infectious disease?

In COVID-19, hypocalcemia is highly prominent, being reported in 60% or more of patients at hospital admission [68–70]. It is associated with hospitalization itself (as the strongest of nine risk factors reported in [68]), longer hospitalizations [70], and ventilation, ICU admission, and mortality [69]. The degree of hypocalcemia thus represents a robust metric of disease aggressiveness [68, 69], and calcium supplementation has been suggested [68, 71].

A decision to treat hypocalcemia, in COVID-19 or other infectious diseases, is based on the assumption that this physiological phenotype is more deleterious for the patient than are normal levels of calcium. This assumption is unwarranted without further evidence. Hypocalcemia, like many other signs and symptoms of a disease state, may represent either: (i) a pathological effect of disease that is beneficial to neither the virus nor the host; (ii) a tactic of the virus to enhance its own growth, survival and transmission, that is deleterious to the host; or (iii) a defence of the host against the virus, that is instigated by the host to make the environment of the virus less hospitable [72, 73]. In this latter case, hypocalcemia would exert negative physiological effects on the host, but would be relatively more deleterious for the virus, providing a net benefit to hosts in terms of survival [8]. Under this scenario, the degree of hypocalcemia is also expected to be positively associated with disease severity, as is typically observed [68, 69, 73]. Hypocalcemia also tends to resolve spontaneously among hospitalized patients [74], as predicted if it represents a conditionally adaptive state. Finally, the defence hypothesis predicts that hypocalcemia is induced by the host, rather than by the virus.

What are the implications of these hypotheses for therapy? By hypothesis (1) above, replacement of serum calcium should tend to normalize host physiology and generate better disease outcomes. Under hypothesis (2), low serum calcium is beneficial to the virus, so replacing calcium should harm the virus, again improving outcomes. But under hypothesis (3), calcium replacement should be harmful to the host, or neutral if the body can still sustain a low-calcium equilibrium [73]. For COVID-19, data are not available on the effects of calcium supplementation in hypocalcemic patients. However, in other cases of critical illness, calcium supplementation has been shown to increase mortality rates, in humans [75–77] and in animal models (e. g. [78, 79]). There is also no evidence that calcium supplementation reduces mortality among patients in the ICU [73, 80].

Evolved host defence tactics that harm the self, as well as a pathogen, carry risks in that, for any given patient with severe disease, the defence can become sufficiently pronounced to increase morbidity or mortality [8]. In such situations, clinical decisions regarding treatment need to become more nuanced. For example, in a retrospective study of patients with sepsis, He *et al*. [77] found the lowest mortality among individuals with mild hypocalcemia, which they considered as protective. These patients were harmed by calcium supplementation. In contrast, patients with severe hypocalcemia showed benefits from supplementation, as expected if their defence system was in these cases imposing undue costs. Prospective clinical trials are needed for robust tests of the costs and benefits of calcium supplementation in patients with different degrees of hypocalcemia.

Cases of extreme and deleterious host defences, such as a substantial level of hypocalcemia, are also not unexpected, given that: (i) levels of expression of such defences should be adapted to ancestral human environments, and to the range and intensity of pathogens to which humans were formerly exposed, and (ii) some pathogens, such as SARS-CoV-2, are novel to humans such that some degree of initial host-pathogen adaptive mismatch is expected. As such, a substantial degree of hypocalcemia in COVID-19 may, like high levels of inflammation, be excessive and deleterious in many patients.

The important question then becomes, if CCBs, and hypocalcemia, may be beneficial against COVID-19 at least in some cases, then might their physiological effects be similar or the same? Do both CCBs and hypocalcemia interfere with the calcium metabolism of pathogens, and thereby inhibit their replication? Hypocalcemia, and CCBs, have, as noted above, both been reported to mediate reductions in intracellular calcium [37, 81], and amlodipine reduces intracellular calcium (in neurons) in a cellular model of Batten disease [82]. The effects of CCBs and hypocalcemia in COVID-19 require targeted studies that take account of host-pathogen conflicts over calcium, and the possibility that hypocalcemia represents a conditional host defence rather than a unilaterally deleterious state.

## FUTURE DIRECTIONS

The decision of whether and how to manage hypocalcemia in COVID-19 patients remains an open question deserving further study. Hypocalcemia is associated with disease severity in COVID-19, prompting some clinicians to advocate for calcium supplementation [68, 71]. However, hypocalcemia may well be protective, as suggested by the evidence described above, in which case calcium supplementation will not improve COVID-19 outcomes, and may indeed worsen them. Since equipoise exists in deciding whether to treat low calcium in COVID-19, calcium supplementation in COVID-19 patients should occur in the setting of well-designed randomized controlled trials.

Evidence from retrospective observational trials, and a recent phenome-wide association study, point to the therapeutic potential of CCBs in general, and amlodipine in particular, against COVID-19. Recent preclinical work also suggests that the CCB diltiazem, in conjunction with remdesivir, may provide notable benefits [83]; this CCB, and others, are in clinical trials for COVID-19 efficacy. Diltiazem is also effective against several other viruses, including a porcine coronavirus (Table 1). Large scale prospective trials will be useful in answering the question of whether these and other CCBs have protective effects for those taking these medications chronically. Whether to initiate CCBs for patients with COVID-19 is another unanswered question. In this context, the potential benefits of starting CCBs should be weighed against their potential harms, including lowering blood pressure and inhibiting hypoxic pulmonary vasoconstriction, which might negatively affect oxygenation during pneumonia [84]. This hypothesis requires direct tests in cellular and animal models, as well as trials in human patients.

## CONCLUSIONS

The development of effective therapies for COVID-19 will benefit from several evolutionary medical considerations, including the evolutionary dynamics and mechanisms of host–virus conflicts over calcium, the recognition that some symptoms of the disease may represent host defences rather than pathologies or viral adaptations, and the fact that SARS-CoV-2 is novel to humans, such that maladaptive phenotypes are not unexpected in both viruses and hosts. Calcium ions are central to coronavirus replication, and available evidence suggests that both CCBs, and hypocalcemia, may interfere with viral replication by reducing levels of intracellular calcium.
